# Confronting antimicrobial resistance in Jordan: regulatory, economic, and behavioral determinants of non-prescription antibiotic dispensing in community pharmacies—a mixed-methods study

**DOI:** 10.3389/fmed.2025.1742205

**Published:** 2026-01-12

**Authors:** Anas Abed, Mohammad Abu Assab, Zekrayat J. H. Merdas, Wael Abu Dayyih, Majd Nawras Maaita, Zainab Zakaraya, Badriyah S. Alotaibi, Nawal Alsubaie

**Affiliations:** 1 Department of Biopharmaceutics and Clinical Pharmacy, Faculty of Pharmacy, Al-Ahliyya Amman University, Amman, Jordan; 2 Clinical Pharmacy Department, Faculty of Pharmacy, Zarqa University, Zarqa, Jordan; 3 Pharmacy Department, College of Pharmacy, Amman Arab University, Amman, Jordan; 4 Faculty of Pharmacy, Mutah University, Al-Karak, Jordan; 5 Department of Pharmaceutical Sciences, College of Pharmacy, Princess Nourah bint Abdulrahman University, Riyadh, Saudi Arabia; 6 Department of Pharmacy Practice, College of Pharmacy, Princess Nourah bint Abdulrahman University, Riyadh, Saudi Arabia

**Keywords:** antimicrobial resistance, antimicrobial stewardship, community pharmacists, Jordan, mixed methods, over-the-counterantibiotics

## Abstract

**Background:**

Over-the-counter (OTC) antibiotic dispensing remains a persistent challenge in many low- and middle-income countries despite the implementation of antimicrobial stewardship (AMS) policies. Jordan's community pharmacies represent a critical interface between public health and antimicrobial resistance (AMR) control. This study aimed to examine the determinants, barriers, and enabling factors influencing AMS adherence among Jordanian community pharmacists, and to integrate quantitative and qualitative evidence to develop a contextual framework for sustainable stewardship implementation.

**Methods:**

A convergent mixed-methods design was employed between March and August 2025. The quantitative phase comprised a cross-sectional survey of 348 community pharmacists recruited through stratified random sampling across five Jordanian governorates. A validated Arabic questionnaire assessed perceived barriers, determinants, and enabling factors related to AMS. Descriptive, non-parametric, and multivariate ordinal logistic regression analyses were conducted, with diagnostic checks for model assumptions, multicollinearity, and goodness-of-fit. The qualitative phase involved 24 semi-structured interviews analyzed using Braun and Clarke's thematic approach with NVivo version 14. Data integration followed Creswell and Plano Clark's parallel design to identify convergence, complementarity, and divergence across datasets.

**Results:**

Pharmacists demonstrated strong awareness of AMS principles, yet significant barriers persisted at structural, social, and economic levels. The most commonly reported barriers included patient pressure for antibiotics, weak regulatory enforcement, and economic dependency on sales. Predictors of higher perceived barriers included working in independent pharmacies, having less than 5 years of experience, and practicing in rural settings. Attendance at AMS or continuing professional development training was protective. Qualitative findings reinforced these results, revealing five themes: regulatory and enforcement gaps, patient-driven and cultural pressures, economic constraints, limited AMS training, and policy improvement recommendations. Integration of both components (qualitative and quantitative) highlighted that pharmacists' willingness to comply with AMS is constrained by system-level weaknesses rather than attitudinal deficits.

**Conclusion:**

Antimicrobial stewardship in Jordan's community pharmacies is hindered primarily by inconsistent enforcement and economic pressures rather than lack of professional awareness. Strengthening regulatory oversight, implementing digital prescription verification, and integrating AMS education into continuing professional development and pharmacy curricula are essential to achieve sustained behavioral change. The integrated conceptual model proposed by this study offers a policy-informed roadmap to strengthen community-based stewardship within similar health system contexts.

## 1 Introduction

Antimicrobial resistance (AMR) represents one of the most urgent global public health threats of the twenty-first century (1). The misuse and overuse of antibiotics in both human and animal health have accelerated the emergence of resistant pathogens, undermining the efficacy of essential medicines and jeopardizing progress in infectious disease control (2). The World Health Organization (WHO) estimates that, without decisive action, AMR could cause up to 10 million deaths annually by 2050 and impose a global economic burden of USD 100 trillion (3). In low- and middle-income countries (LMICs), the inappropriate use of antibiotics is often driven by the ease of over-the-counter (OTC) access, weak regulatory enforcement, and economic incentives within private pharmacy sectors (4). Consequently, the WHO has emphasized the need for community-based antimicrobial stewardship (AMS) programs as a cornerstone of its Global Action Plan on AMR (2015–2030) (5).

In Jordan, antibiotics remain widely accessible without prescription despite legal restrictions under the national Pharmacy Law No. 12 (2013), which mandates prescription-only sales (6). Studies conducted over the past decade consistently report high rates of non-prescription antibiotic dispensing by community pharmacists. For instance, Almaaytah et al. (7) found that approximately 74.3% of Jordanian pharmacies sold antibiotics without a prescription, primarily for upper respiratory tract infections and urinary complaints. Similarly, Haddadin et al. (8) observed that more than two-thirds of antibiotics dispensed in retail pharmacies were OTC, often for self-limiting conditions. More recent investigations reaffirm that despite increasing awareness of AMR, the practice remains deeply entrenched due to patient expectations, profit pressures, and inconsistent enforcement of regulations (9–11).

These studies collectively highlight a paradox within Jordan's pharmacy sector where pharmacists are generally aware of antimicrobial resistance and stewardship principles but continue to dispense antibiotics without prescriptions. The reasons for this discrepancy, however, remain insufficiently explored. While prior Jordanian research has provided valuable insights into community pharmacists' antibiotic dispensing practices, awareness of antimicrobial resistance, and perceived AMS barriers, these studies rely predominantly on single-method approaches, either quantitative surveys assessing rates or qualitative interviews exploring perceptions, which limit the ability to link measurable predictors with underlying behavioral mechanisms. Similar methodological limitations are also evident in regional studies across the Middle East. This study extends the literature by employing a convergent mixed-methods design that integrates a large-scale quantitative survey (*n* = 348) with in-depth qualitative interviews. This approach enables us to (1) identify independent predictors of high perceived AMS barriers using multivariate ordinal logistic regression; and (2) contextualize these predictors through thematic narratives of pharmacists' lived experiences. The integration of quantitative and qualitative components, following Creswell and Plano Clark's parallel design, yields a contextualized behavioral model for AMS adherence in Jordanian community pharmacies, offering an extension and deepening of existing work to inform policy and practice.

## 2 Methods

### 2.1 Study design and overview

This study employed a convergent mixed-methods design, combining a quantitative cross-sectional survey with qualitative semi-structured interviews. This approach enabled data triangulation, with the quantitative phase identifying measurable determinants and barriers to AMS and the qualitative phase offering deeper insights into pharmacists' lived experiences and contextual challenges. Data from both phases were collected sequentially, analyzed separately, and integrated during interpretation to develop a unified conceptual model of stewardship behavior.

### 2.2 Setting and participants

The study was conducted across five major Jordanian governorates—Amman, Irbid, Zarqa, Balqa, and Karak—to capture a balance of urban and rural pharmacy settings. The target population comprised all licensed community pharmacists who are actively practicing during the study period. Eligibility required at least 6 months of practice and provision of digital consent; pharmacists working exclusively in hospital, industrial, or academic roles were excluded.

For the quantitative phase, stratified random sampling ensured proportional representation from independent and chain pharmacies. In the qualitative phase, purposive sampling targeted maximal variation in gender, years of experience, and location.

### 2.3 Sample size and power calculation

The quantitative sample size was calculated using Cochran's formula for categorical data, assuming a 70% expected prevalence of non-prescription antibiotic dispensing (7), a 95% confidence level, and a 5% margin of error. This yielded a minimum of 323 pharmacists, and 348 complete responses were obtained.

For the qualitative phase, 24 pharmacists participated in semi-structured interviews. Thematic saturation was reached when no new codes emerged across two consecutive interviews (confirmed after interview 22), with two additional interviews performed to verify redundancy.

### 2.4 Instrument development, translation, and validation

The quantitative questionnaire was adapted from previously validated AMS tools used in Gulf and Middle Eastern contexts (12–14), then contextualized for Jordan via expert consultation. Initially drafted in English, it was translated into Arabic using a forward–backward protocol by two independent bilingual translators, with back-translation performed blindly. A reconciliation meeting involving the research team and language experts confirmed semantic, idiomatic, and conceptual equivalence.

Cross-cultural validity was established through a pilot with 30 community pharmacists, assessing clarity, cultural relevance, and psychometric properties. Exploratory factor analysis showed factor loadings >0.6, and Cronbach's α ranged from 0.86 to 0.91, indicating excellent internal consistency. The Arabic version was thus deemed linguistically and psychometrically valid.

The final instrument included four domains: (1) demographic and practice characteristics; (2) perceived barriers to prescription-only antibiotic dispensing; (3) enabling and determinant factors of AMS compliance; and (4) attitudes toward AMR. All items used a five-point Likert scale (“strongly disagree” to “strongly agree”).

The qualitative interview guide comprised six open-ended core questions with probing prompts, exploring: (1) daily experiences and patient behavior regarding antibiotic requests without prescription; (2) barriers to adherence to prescription-only dispensing; (3) perceptions of regulatory enforcement and inspections by the Ministry of Health (MoH); (4) understanding and application of AMS principles, including prior training and confidence in patient counseling; (5) economic and organizational influences on dispensing behavior; and (6) recommendations for policy, regulatory, and educational improvements. A pilot with two pharmacists refined question clarity, flow, and cultural appropriateness.

### 2.5 Data collection procedures

Quantitative data were gathered from March to June 2025 via a self-administered online questionnaire distributed through social media platforms. Participants received an electronic information sheet detailing study objectives, procedures, and confidentiality safeguards, followed by digital informed consent. Responses were anonymous, with an average completion time of 10–12 min. No personal identifiers were collected.

Qualitative data collection occurred from July to August 2025, with semi-structured interviews in Arabic conducted by the principal investigator either face-to-face or via secure Zoom, based on participant preference and accessibility. Sessions lasted 35–45 min and were audio-recorded using encrypted devices after explicit consent. Transcripts were produced verbatim within 48 h, verified against recordings for accuracy, and analyzed in Arabic to retain linguistic and cultural nuances; English translations were cross-checked by bilingual pharmacists for semantic and conceptual fidelity. Field notes captured contextual details and non-verbal observations immediately after each interview.

To minimize insider bias and ensure interviewer neutrality, several measures were implemented. The interviewer maintained a reflexive journal throughout the data-collection period, documenting preconceptions (e.g., anticipated emphasis on economic pressures) and consciously using open, non-directive probes such as “Can you tell me more about that situation?” or “How did that make you feel?”. A co-investigator based outside Jordan independently reviewed 20% of randomly selected transcripts for evidence of leading questions; no systematic bias was identified. Anonymity was repeatedly emphasized, and interviews began with neutral topics about daily pharmacy practice before progressing to antibiotic-related issues, thereby reducing social-desirability bias.

Member-checking was performed by sending a one-page plain-language summary of the emerging themes to eight participants (33% of the qualitative sample). Seven responded, and confirmed that the summary accurately reflected their views, with no requests for major changes.

### 2.6 Data analysis

Quantitative analysis used Prism 9.0 software. Descriptive statistics included medians with interquartile ranges (IQR) for ordinal data and frequencies with percentages for categorical variables. Group differences were assessed via Mann–Whitney *U* and Kruskal–Wallis tests. Variables with *p* < 0.10 in bivariate analyses entered a multivariate ordinal logistic regression model to identify independent predictors of high perceived barriers. Composite scores for both barrier and enabling domains were calculated by averaging participants' responses across each set of Likert-scale items (1 = strongly disagree to 5 = strongly agree). Higher barrier scores indicated greater perceived difficulty in enforcing prescription-only antibiotic dispensing, whereas higher enabling scores reflected stronger agreement that the corresponding factors facilitated stewardship adherence. Both domains demonstrated excellent internal consistency (Cronbach's α > 0.85). Model assumptions, multicollinearity (variance inflation factor < 5), and goodness-of-fit (Brant test) were verified.

Qualitative analysis followed Braun and Clarke's six-phase reflexive thematic analysis framework using the NVivo 14. The first 12 transcripts were independently double-coded inductively by two independent researchers, yielding a Cohen's kappa of 0.83 for inter-coder agreement. Coding discrepancies, which primarily involved differing interpretations of “patient pressure” as cultural vs. economic, were resolved through consensus discussions with a third investigator acting as arbiter. A final codebook was agreed upon and applied to the full dataset, after which themes and sub-themes were audited by the entire author team. A cumulative coding log was maintained to track the emergence of new codes; no new codes appeared after the 22nd interview.

**Figure 1:**
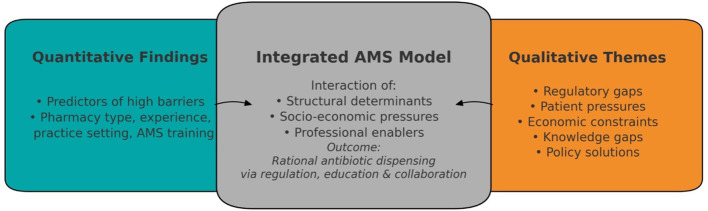
The integrated conceptual model derived from the mixed-methods analysis. Quantitative results **(left)** identify measurable predictors of high barrier perception, while qualitative insights **(right)** explain the underlying mechanisms through five emergent themes. The central layer represents the dynamic interaction between these structural, economic, and cultural factors, which collectively shape pharmacists' dispensing behavior.

Following Creswell and Plano Clark's convergent parallel design, quantitative and qualitative analyses were conducted independently before merging via a joint display matrix that aligned statistical results with themes. This process identified convergence, complementarity, and divergence, with qualitative narratives contextualizing quantitative trends. The integration equally weighted prevalence and meaning, informing a conceptual framework (Figure 1, Section 3.6) that illustrates interactions among structural, socio-economic, and professional determinants shaping antibiotic-dispensing behavior.

### 2.7 Ethical considerations

Ethical approval was granted by the Institutional Review Board of Al-Ahliyya Amman University (IRB No. AAU/3/7/2024-2025). Participation was voluntary, with informed consent secured prior to enrolment. Confidentiality was maintained by excluding personal identifiers and storing data on encrypted, password-protected drives accessible only to the research team. The study fully complied with the Declaration of Helsinki.

## 3 Results

As elucidated in Table 1, A total of 348 community pharmacists across Jordan completed the survey (response rate = 87%), providing broad regional representation, with Amman contributing the largest share. Most respondents were female and in their early thirties. Nearly half were aged 25–34 years, and more than three-quarters held either a BPharm or PharmD degree.

**Table 1:** Sociodemographic characteristics of community pharmacists in Jordan (*n* = 348).

**Variable**	**Category**	***n* (%)**
Gender	Male	123 (35.3)
	Female	225 (64.7)
Age (years)	< 25	77 (22.1)
	25–34	165 (47.4)
	35–44	75 (21.6)
	≥45	31 (8.9)
Qualification	BPharm	200 (57.5)
	PharmD	124 (35.6)
	Postgraduate	24 (6.9)
Experience (years)	< 5	99 (28.4)
	5–10	136 (39.1)
	11–15	77 (22.1)
	>15	36 (10.4)
Pharmacy type	Independent	237 (68.1)
	Chain	111 (31.9)
Setting	Urban	251 (72.1)
	Rural	97 (27.9)
Main patient base	Insured	192 (55.2)
	Uninsured/self-pay	156 (44.8)
Attended AMS training (past 2 years)	Yes	196 (56.3)
Familiar with National AMR Plan	Yes	103 (29.6)

Over two-thirds worked in independent pharmacies, and about three-quarters practiced in urban areas. Professional experience was well distributed, though fewer than one-third had practiced for more than 10 years. Almost half reported serving predominantly uninsured patients, reflecting socioeconomic diversity across practice settings. In terms of stewardship exposure, just over half had attended AMS-related training in the past 2 years, yet fewer than one-third were aware of Jordan's National Action Plan for Antimicrobial Resistance (2018–2025).

### 3.1 Perceived barriers to restricting over-the-counter antibiotic sales

Pharmacists reported several interconnected challenges that hinder adherence to prescription-only antibiotic dispensing (Table 2). Overall, many pharmacists perceive difficulty in enforcing existing regulations, although the magnitude of differences across groups was modest. The most frequently cited obstacles were patient insistence, weak enforcement of laws, and low public awareness of antimicrobial resistance. Perceived economic pressures related to maintaining pharmacy income and customer satisfaction were frequently described as compounded these difficulties.

**Table 2:** Perceived barriers to restricting over-the-counter antibiotic sales among community pharmacists in Jordan (*n* = 348).

**Barrier item**	**Median [IQR]**	**% Agree/strongly agree**	***p*-value (by pharmacy type)**
High patient demand and pressure to dispense antibiotics	4.6 [4.0–5.0]	89.7	0.011
Weak enforcement of pharmacy regulations	4.5 [4.0–5.0]	85.9	0.026
Lack of public awareness about antibiotic misuse	4.3 [3.8–4.9]	83.2	0.214
Economic pressure to maintain sales and customer satisfaction	4.1 [3.4–4.6]	79.6	0.002
Lack of regular pharmacy inspection by MoH	3.9 [3.2–4.5]	75.8	0.034
Absence of digital prescription verification system	3.7 [3.0–4.2]	71.0	0.121
Limited collaboration with physicians regarding antibiotic requests	3.6 [3.0–4.1]	68.4	0.087
Fear of losing customers to competing pharmacies	3.5 [2.9–4.3]	66.7	0.019
Inadequate professional training on antimicrobial stewardship	3.3 [2.8–4.0]	63.8	0.043
Lack of time for patient counseling	3.1 [2.5–3.9]	57.2	0.065

*p*-values obtained using Kruskal–Wallis test comparing independent vs. chain pharmacies.

Other commonly mentioned barriers included infrequent Ministry of Health inspections, the absence of digital prescription verification systems, and limited collaboration with physicians. Together, these findings point to multiple interacting factors that may influence pharmacists' experiences, though the results do not quantify their relative contributions.

Notably, independent pharmacies reported greater challenges related to patient pressure and financial constraints compared with chain pharmacies, likely reflecting differences in oversight and business models. Similarly, less-experienced pharmacists expressed more difficulty refusing non-prescription requests than their senior counterparts, suggesting that confidence in ethical decision-making strengthens with professional maturity.

### 3.2 Determinants and enabling factors for antimicrobial stewardship

Despite the considerable barriers identified, pharmacists also reported several factors that supported adherence to AMS principles (Table 3). Overall, enabling factor scores reflected favorable attitudes toward stewardship when structural and educational support were present. Participation in AMS programs or continuing professional development (CPD) training emerged as the strongest facilitator, with trained pharmacists expressing greater confidence in refusing inappropriate requests and providing patient counseling (*p* = 0.009). Similarly, Pharmacists familiar with Jordan's National Action Plan for Antimicrobial Resistance (2018–2025) tended to report fewer perceived difficulties in stewardship practice, underscoring the importance of policy awareness in shaping behavior.

**Table 3:** Determinants and enabling factors influencing antimicrobial stewardship among community pharmacists in Jordan (*n* = 348).

**Determinant/enabling factor**	**Median [IQR]**	**% Agree/strongly agree**	***p*-value (by pharmacy type)**
Participation in AMS/CPD training enhances compliance	4.6 [4.2–5.0]	87.9	0.009
Awareness of the National AMR Action Plan improves adherence	4.4 [3.8–4.9]	82.1	0.021
Collaboration with physicians supports appropriate antibiotic use	4.2 [3.6–4.7]	79.8	0.035
Clear and consistent MoH regulations promote compliance	4.1 [3.5–4.5]	76.7	0.028
Regular pharmacy inspection encourages rational dispensing	4.0 [3.3–4.5]	74.3	0.045
Availability of digital prescription systems would facilitate AMS	3.9 [3.3–4.4]	70.1	0.067
Supportive workplace policies (chain pharmacy protocols) enhance compliance	3.8 [3.1–4.3]	69.0	0.017
Ethical commitment and professional responsibility are key motivators	4.3 [3.9–4.8]	84.2	0.140

*p*-values obtained using Kruskal–Wallis test comparing independent vs. chain pharmacies.

Collaboration with physicians was identified as an additional factor linked to more favorable stewardship experiences. Pharmacists noted that cooperative communication with prescribers strengthened their confidence during patient counseling and reduced perceived conflict when refused non-prescription antibiotic requests. Collaboration scores were higher among pharmacists in urban settings (*p* = 0.035), this pattern may be related to closer proximity to healthcare facilities and more established interprofessional networks.

Perceived regulatory clarity corresponded to more favorable stewardship perceptions and should be interpreted cautiously. pharmacists who perceived Ministry of Health regulations as clear and consistently enforced reported lower perceived barriers to prescription-only antibiotic dispensing (*p* = 0.028). Conversely, inconsistent inspection practices were frequently described as reducing motivation to adhere strictly to dispensing regulations. Many participants also expressed optimism that introducing digital prescription verification systems could improve accountability and support regulatory implementation.

Workplace structure further influenced stewardship behavior. Chain pharmacy pharmacists reported reported marginally fewer perceived challenges related to stewardship practice than those in independent settings (*p* = 0.017), attributing this to internal supervision and standardized protocols. Nonetheless, across all groups, respondents consistently highlighted ethical commitment and professional responsibility were frequently cited as intrinsic motivators, though their relative influence was not quantified.

### 3.3 Associations between demographic variables and perceived barriers

Statistical analysis was conducted to explore the association between pharmacists' demographic and professional characteristics and their perceived barriers to restricting over-the-counter antibiotic sales. The overall model revealed several significant relationships, indicating that the perception of barriers is influenced by both individual and workplace factors (Table 4).

**Table 4:** Associations between demographic variables and perceived barriers to restricting over-the-counter antibiotic sales (*n* = 348).

**Variable**	**Category**	**Median barrier score [IQR]**	***p*-value**
Gender	Male	3.9 [3.3–4.5]	0.267
	Female	4.0 [3.4–4.5]	
Age (years)	< 25	4.2 [3.7–4.8]	0.024
	25–34	3.9 [3.3–4.5]	
	35–44	3.7 [3.1–4.3]	
	≥45	3.6 [3.0–4.1]	
Experience (years)	< 5	4.3 [3.7–4.8]	0.018
	5–10	3.9 [3.4–4.4]	
	11–15	3.6 [3.1–4.2]	
	>15	3.5 [3.0–4.0]	
Pharmacy type	Independent	4.1 [3.6–4.7]	0.012
	Chain	3.5 [3.0–4.1]	
Practice setting	Urban	3.8 [3.2–4.3]	0.041
	Rural	4.2 [3.6–4.8]	
Educational qualification	BPharm	4.0 [3.4–4.5]	0.133
	PharmD/ Postgraduate	3.8 [3.3–4.3]	

*p*-values obtained using the Kruskal–Wallis test for ordinal comparisons.

Pharmacists with less than 5 years of experience reported significantly higher barrier scores compared to those with more than 10 years of practice (*p* = 0.018). Early-career pharmacists frequently cited difficulty in managing patient expectations and a lack of confidence in refusing non-prescription antibiotic requests. This finding suggests that clinical experience and professional maturity are associated with slightly lower perceived barriers to applying stewardship principles.

Pharmacy ownership type also had a notable influence. Those working in independent pharmacies perceived more intense barriers than pharmacists in chain pharmacies (*p* = 0.012). This difference may reflect the absence of structured oversight or internal accountability systems in independent settings, where individual pharmacists often bear the full burden of enforcing prescription-only regulations while maintaining business viability.

Geographical location showed additional variation. Pharmacists practicing in rural or underserved areas reported higher barrier scores (*p* = 0.041), which may be partly related to greater patient reliance on pharmacies as first-line healthcare access points. In these settings, pharmacists described the refusal of antibiotic requests as socially and economically challenging, particularly in communities where physician consultation is less accessible or affordable.

No statistically significant differences were observed by gender (*p* = 0.267) or educational qualification (*p* = 0.133), although pharmacists holding PharmD degrees generally exhibited slightly lower barrier scores, possibly reflecting stronger clinical training and stewardship awareness.

### 3.4 Predictors of high perceived barriers

To further explore the determinants of pharmacists' perceived barriers, a multivariate ordinal logistic regression model was performed. The dependent variable was the overall barrier score, dichotomized at the median to distinguish between high and low-to-moderate barrier perception. Independent variables included gender, age, years of experience, pharmacy type, practice setting, AMS training attendance, and familiarity with the national AMR plan.

The final model (Nagelkerke *R*^2^ = 0.32, *p* < 0.001) identified pharmacy type, years of experience, and practice setting as significant predictors of high perceived barriers (Table 5). Pharmacists working in independent pharmacies were more than twice as likely to report high barrier scores compared with those employed in chain pharmacies (AOR = 2.34, *p* = 0.001), although the overall effect size remained moderate. Early-career practitioners also demonstrated higher odds of reporting elevated perceived difficulty (AOR = 1.89, *p* = 0.017), indicating differences in perceived barriers rather than confirmed behavioral non-compliance.

**Table 5:** Ordinal logistic regression model for predictors of high perceived barriers to restricting over-the-counter antibiotic sales among community pharmacists in Jordan (*n* = 348).

**Predictor variable**	**Adjusted odds ratio (AOR)**	**95% Confidence interval**	***p*-value**
Gender (female vs. male)	1.12	0.71–1.76	0.621
Age (< 35 vs. ≥35 years)	1.21	0.73–2.03	0.463
Experience (< 5 vs. ≥5 years)	1.89	1.12–3.17	0.017
Pharmacy type (independent vs. chain)	2.34	1.41–3.86	0.001
Practice setting (rural vs. urban)	1.76	1.04–2.98	0.034
Attended AMS/CPD training (yes vs. no)	0.63	0.41–0.97	0.039
Aware of National AMR Plan (yes vs. no)	0.74	0.46–1.18	0.203
Educational qualification (PharmD/Postgraduate vs. BPharm)	0.91	0.53–1.56	0.735

Model statistics; Nagelkerke R^2^ = 0.32; model χ^2^(8) = 42.5, *p* < 0.001.

Rural practice location emerged as another independent predictor (AOR = 1.76, *p* = 0.034), which may be related to reduced healthcare access and stronger patient reliance on pharmacies for immediate treatment. In contrast, attendance at AMS or CPD training coincided with comparatively lower odds of perceiving high barriers (AOR = 0.63, *p* = 0.039), indicating the protective influence of educational exposure. Awareness of the National AMR Plan demonstrated a similar, though non-significant, inverse relationship (AOR = 0.74, *p* = 0.203).

Gender, age, and educational qualification were not statistically significant predictors after controlling for other variables.

### 3.5 Qualitative findings

A total of 24 community pharmacists participated in semi-structured interviews that explored their experiences with OTC antibiotic sales, perceptions of enforcement mechanisms, and views on AMS. Participants represented diverse geographic regions and practice settings, with data saturation achieved after 22 interviews. Thematic analysis identified five major themes: (1) regulatory and enforcement gaps; (2) patient-driven and cultural pressures; (3) economic and organizational constraints; (4) professional knowledge and stewardship awareness; and (5) pharmacists' recommendations for policy and practice improvement (Table 6). To avoid redundancy, a single illustrative quotation is provided for each theme, with thematic interpretations derived from recurring patterns across interviews.

**Table 6:** Summary of qualitative themes and representative quotations from interviews with community pharmacists in Jordan (*n* = 24).

**Main theme**	**Subthemes**	**Representative quotations**	**Interpretation**
1. Regulatory and enforcement gaps	• Infrequent MoH inspections • Lack of digital prescription verification • Minimal penalties for violations	“*Inspectors come once a year, sometimes only to check licenses, not prescriptions. Everyone knows the rules, but no one checks compliance*.” (P7, Amman)	Weak and inconsistent enforcement undermines compliance and enables continued OTC antibiotic sales.
2. Patient-driven and cultural pressures	• Patients demand antibiotics for minor illnesses • Cultural belief in antibiotics as “quick cures” • Fear of losing customers	“*People trust pharmacists more than doctors for small problems. They come asking directly for Augmentin or azithromycin like it's Panadol*.” (P4, Zarqa)	Cultural norms and patient expectations place pharmacists under sustained pressure to dispense antibiotics without prescriptions.
3. Economic and organizational constraints	• Financial pressure to maintain sales • Competitive market dynamics • Chain pharmacies offer limited managerial protection	“*You can't afford to lose customers. If I say no, they'll go to another pharmacy next door*.” (P3, Amman)	Economic fragility and absence of incentive mechanisms discourage AMS adherence; particularly in independent pharmacies.
4. Professional knowledge and stewardship awareness	• Limited AMS training exposure • Desire for practical, accessible education • Greater confidence among trained pharmacists	“*We learned about resistance in university, but not how to talk to patients about it. We need real guidance, not just theory*.” (P14, Zarqa)	Gaps in applied AMS training affect pharmacists' confidence in counseling patients and refusing inappropriate antibiotic requests.
5. Recommendations for policy and practice improvement	• Stronger MoH oversight • Digital prescription tracking • Interprofessional collaboration • National awareness campaigns	“*Regulation alone won't solve it. Patients must understand the risks*.” (P10, Balqa)	Pharmacists advocate for integrated interventions combining education, regulation, and public engagement, to support sustainable AMS implementation.

Thematic saturation was achieved after the 22nd interview, with no new codes or subthemes emerging; two additional interviews confirmed redundancy.

#### 3.5.1 Regulatory and enforcement gaps

Pharmacists consistently described weak regulatory enforcement as a central factor sustaining the non-prescription sale of antibiotics. Although all participants were aware that antibiotic dispensing without a prescription is prohibited by law, most reported that inspections by the MoH were infrequent, brief, and largely symbolic. Several pharmacists referred to the regulations as “paper laws” that exist without consistent monitoring or consequences.

“*Inspectors come once a year, sometimes only to check licenses, not prescriptions. Everyone knows the rules, but no one checks compliance*.” (Pharmacist 7, Amman)

Many participants also pointed out the lack of a unified digital prescription tracking system, which makes it difficult to verify whether antibiotics were prescribed legitimately. Pharmacists viewed the absence of digitalization as a major regulatory blind spot that enables patients to move between pharmacies until they find one willing to sell antibiotics.

#### 3.5.2 Patient-driven and cultural pressures

A second major theme concerned patient expectations and cultural norms surrounding antibiotic use. Pharmacists reported that antibiotics are commonly perceived as quick solution for common minor ailments such as sore throats, colds, or flu-like symptoms, placing them in ethically challenging situations, particularly when patients become insistent or emotionally persuasive.

“*People trust pharmacists more than doctors for small problems. They come asking directly for Augmentin or azithromycin like it's Panadol*.” (Pharmacist 4, Zarqa).

Pharmacists in rural and underserved areas faced stronger patient pressure, where limited access to physicians and consultation costs often justified antibiotic self-medication. These encounters created a moral tension between maintaining professional standards and sustaining good customer relationships essential for business continuity.

#### 3.5.3 Economic and organizational constraints

Economic influences discussed in this section reflect pharmacists' self-reported perceptions and experiences rather than objectively measured financial indicators.

Economic pressures emerged as a recurring theme, particularly among pharmacists working in independent community pharmacies. Participants explained that the profit margins on antibiotics and the need to retain customers make it difficult to enforce strict prescription rules.

“*You can't afford to lose customers. If I say no, they'll go to another pharmacy next door*.” (Pharmacist 3, Amman)

By contrast, pharmacists employed in chain pharmacies noted that corporate protocols provided some protection, as refusal decisions were supported by management and documented in internal reporting systems. However, even within chains, the pressure of sales targets and commercial competition occasionally undermined adherence to AMS principles.

#### 3.5.4 Professional knowledge and stewardship awareness

While most participants recognized the concept of antimicrobial resistance, many admitted that their practical understanding of stewardship strategies was limited. Only a few pharmacists reported receiving formal AMS training during their studies or through continuing education workshops.

“*I learned about resistance in university, but not how to talk to patients about it. We need real guidance, not just theory*.” (Pharmacist 14, Zarqa)

Those who had participated in AMS or CPD programs expressed greater confidence in handling patient requests and reported stronger commitment to responsible antibiotic use. Several participants highlighted the need for accessible, accredited AMS courses integrated into pharmacy licensing and renewal requirements.

#### 3.5.5 Pharmacists' recommendations for policy and practice improvement

Participants offered concrete and often pragmatic recommendations for reducing non-prescription antibiotic sales. The most recurrent suggestions included: (1) strengthening MoH inspection frequency and transparency; (2) implementing a national digital prescription system to prevent pharmacy shopping; (3) providing continuing education programs tailored to community pharmacy realities; and (4) launching public awareness campaigns to shift social norms regarding antibiotic use.

“*Regulation alone won't solve it. Patients must understand the risks. We need national awareness, not just penalties*.” (Pharmacist 10, Balqa)

Pharmacists also called for improved collaboration with physicians, emphasizing that consistent messaging from healthcare professionals would help reduce patient confusion and distrust. The interviews collectively reflected a strong professional willingness to comply, provided that enabling infrastructure and public education mechanisms are in place.

The qualitative findings reveal that pharmacists in Jordan face a complex interplay of regulatory, social, and economic pressures that collectively sustain non-prescription antibiotic sales. Weak law enforcement and economic fragility form the structural backdrop, while patient expectations and limited public awareness create daily interpersonal challenges. Importantly, pharmacists expressed both ethical awareness and professional readiness to adopt stewardship-aligned practices if supported by coherent policies, regular training, and consistent inspection mechanisms.

### 3.6 Integration of quantitative and qualitative findings

The mixed-methods integration demonstrated general convergence between the quantitative patterns and qualitative narratives, confirming that the barriers to antimicrobial stewardship among Jordanian community pharmacists are driven by a combination of structural, social, and economic determinants rather than by lack of professional awareness. The quantitative findings described the distribution of perceived barriers, while qualitative themes provided contextual explanations to aid interpretation. This synthesis is visualized in the integrated conceptual model (Figure 1), which depicts the interplay of predictors, themes, and outcomes shaping rational antibiotic dispensing.

#### 3.6.1 Convergence of key findings

Quantitatively, pharmacists identified patient pressure, weak law enforcement, and economic dependency on sales as the most significant obstacles to responsible antibiotic dispensing. These same barriers were vividly described in the interviews, where pharmacists articulated the moral and commercial tension of balancing patient satisfaction with legal compliance. The high median barrier score among independent pharmacies and early-career pharmacists was mirrored by narratives describing limited managerial protection, competitive market dynamics, and fear of losing customers.

Similarly, the enabling determinants, particularly AMS training, interprofessional collaboration, and clear MoH regulation, aligned closely with qualitative accounts. Pharmacists who had attended stewardship workshops reported stronger confidence and ethical resilience, explaining the lower barrier scores observed quantitatively among trained participants. The interviews enriched this pattern by revealing that pharmacists perceive training as both knowledge reinforcement and psychological empowerment when negotiating with insistent patients.

#### 3.6.2 Expansion and divergence

The qualitative findings expanded the quantitative results by exposing latent systemic weaknesses not fully captured in the survey. While the questionnaire documented “weak enforcement” as a numeric barrier, interviews detailed how regulatory gaps operate, sporadic inspections, lack of penalties, and absence of digital prescription monitoring. The qualitative strand also revealed the cultural normalization of antibiotic use for minor ailments, a dimension that was not directly measured in the quantitative tool but emerged as a powerful social determinant influencing daily pharmacy practice.

A mild divergence was observed in attitudes toward professional responsibility: survey data suggested uniformly high ethical motivation, whereas interviews showed that economic survival often moderates moral idealism, particularly in small independent pharmacies. This divergence highlights that self-reported attitudes may overestimate stewardship compliance when commercial pressure is unacknowledged.

#### 3.6.3 Complementarity and policy synthesis

Together, the quantitative and qualitative components of the study provide a comprehensive explanatory model of antimicrobial stewardship behavior in Jordan (Figure 1). Quantitative results quantify the scale of regulatory and experiential influences, while qualitative insights clarify the causal pathways and emotional realities underlying those statistics. To exemplify this integration per GRAMMS guidelines, Table 7 presents a joint display aligning key quantitative metrics with corresponding qualitative evidence, illustrating convergence (e.g., high patient pressure), complementarity (e.g., explanatory depth on rural challenges), and divergence (e.g., nuanced economic moderation of ethics). This integration underscores that sustainable change requires systemic alignment between education, regulation, and economics:

- Education builds pharmacists' confidence and ethical conviction.
- Regulation ensures accountability through consistent inspection and digital verification.
- Economic and social incentives safeguard business viability while rewarding compliant behavior.

**Table 7:** Joint display of quantitative and qualitative integration.

**Quantitative finding (metric)**	**Qualitative theme (quote)**	**Integration type**	**Interpretation**
Patient pressure: median 4.6 [IQR 4.0–5.0]; 89.7% agree/strongly agree (Table 2); Independent pharmacies higher (*p* = 0.011)	Theme 2: patient-driven pressures “*People trust pharmacists more than doctors for small problems. They come asking directly for Augmentin or azithromycin like it's Panadol*.” (P4, Zarqa)	Convergence	Confirms cultural self-medication norms in Jordan [e.g., for URIs per Almaaytah et al. (7)]; independent settings amplify due to customer retention needs.
Independent pharmacies: AOR = 2.34, 95% CI: 1.41–3.86 (*p* = 0.001) for high barriers (Table 5)	Theme 3: economic constraints “*You can't afford to lose customers. If I say no, they'll go to another pharmacy next door*.” (P3, Amman)	Convergence	Aligns with Jordan's high proportion of independents (68.1%, Table 1); lack of chain oversight exacerbates economic vulnerability in competitive markets.
Rural settings: AOR = 1.76, 95% CI: 1.04–2.98 (*p* = 0.034) for high barriers (Table 5)	Theme 2: patient pressures (expansion) “*If you refuse, they accuse you of arrogance*.” (P12, Karak—rural)	Complementarity	Quantifies rural-urban disparities (27.9% rural sample); qual explains via limited physician access in underserved areas like Karak/Balqa.
AMS training: AOR = 0.63, 95% CI: 0.41–0.97 (*p* = 0.039) protective (Table 5)	Theme 4: knowledge gaps “*We need real guidance, not just theory*.” (P14, Zarqa)	Convergence	Supports integration into Jordan's National AMR Plan (only 29.6% familiar, Table 1); training empowers ethical refusal amid weak MoH enforcement.
Ethical commitment: median 4.3 [IQR 3.9–4.8]; 84.2% agree (Table 3)	Theme 3: economic moderation “*Sometimes I'm torn, I know it's wrong, but I have rent… Refusing costs me loyal customers*.” (P9, Irbid)	Divergence	Survey overestimates uniform ethics; qual reveals economic override in independents, highlighting need for incentives in Jordan's private sector.

This joint display merges prevalence (quant) with meaning (qual) to inform the conceptual model (Figure 1).

The complementary evidence therefore supports a multi-layered strategy: implementing nationwide AMS training programs, enforcing digital prescription codes, and launching culturally sensitive public awareness campaigns. Only through this triangulated approach can the professional readiness identified in both datasets be translated into consistent, population-level reduction in inappropriate antibiotic use.

## 4 Discussion

AMR remains among the gravest threats to global health, projected to cause up to 10 million deaths annually by 2050 if unmitigated (3). Community pharmacists in LMICs like Jordan are frontline actors in AMS, yet our mixed-methods findings expose a critical “knowledge-practice gap” in that despite robust awareness of AMS principles, systemic barriers perpetuate non-prescription antibiotic dispensing, challenging the assumption that professional education suffices to curb misuse (4, 5). This paradox, visualized in our integrated conceptual model (Figure 1), highlights how structural determinants, rather than individual deficits, drive irrational behavior, urging a re-evaluation of AMS strategies beyond awareness campaigns.

In Jordan, antibiotics are not displayed on open shelves or freely accessible within community pharmacies. Instead, they are stored behind the dispensing counter and supplied directly by pharmacists. Non-prescription access typically occurs when patients explicitly request specific antibiotics by name, rather than selecting them independently. These requests are often informed by previous prescriptions, prior personal experience, informal advice from family or peers, or the widespread cultural perception that antibiotics represent a rapid solution for common self-limiting illnesses. This pattern reflects a request-based, rather than self-selection, model of access. Consistent with this, qualitative interviews indicated that patient knowledge of specific antibiotics is largely experiential rather than clinical.

Our results indicate that regulatory inconsistencies and economic incentives undermine even well-intentioned pharmacists, as evidenced by higher barrier perceptions in independent and rural settings. Applying a structure-behavior-policy lens, this suggests that decentralized pharmacy models amplify AMR risks by prioritizing short-term economic survival over ethical dispensing, creating a feedback loop where weak enforcement normalizes non-compliance (15). For instance, the protective effect of AMS training (AOR = 0.63) is evident but limited, revealing a contradiction: while education empowers individual confidence, its behavioral impact falters without supportive infrastructure, such as digital verification systems. This internal tension, between quantitative enabling factors (as presented in Table 3) and qualitative accounts of daily economic pressures, highlights how abstract knowledge translates poorly in resource-constrained environments, contradicting optimistic views in prior LMIC studies that training alone yields sustained change (16, 17).

Patterns in neighboring contexts reinforce yet extend our insights. In Saudi Arabia and Egypt, similar stewardship awareness coexists with persistent dispensing violations, attributed to enforcement lapses and market dynamics (18–21). However, our emphasis on rural-urban disparities (AOR = 1.76 for rural settings) challenges assumptions of uniform barriers across geographies, as qualitative narratives uncover heightened patient reliance in underserved areas, where pharmacies substitute for inaccessible physicians—exacerbating cultural normalization of self-medication. This diverges from urban-centric Palestinian data (21), which downplays location, and implies that blanket regional policies may overlook equity issues, potentially widening AMR disparities. Moreover, the mild divergence in our data, surveys inflating ethical commitment while interviews expose economic moderation of idealism, mirrors social desirability biases in self-reports, questioning the reliability of attitude-based metrics in AMS research (22).

A core contradiction emerges from the interplay of professional readiness and systemic fragility: pharmacists express ethical resolve (high enabling scores), yet economic dependencies temper it, particularly in independent pharmacies (AOR = 2.34). This challenges the prevailing assumption in early LMIC literature that attitudinal shifts via education are the primary lever for change (4, 7–11), as our findings demonstrate structural gaps (e.g., absent digital tracking) as the true bottlenecks. Such evidence disrupts the notion that AMR persistence stems from knowledge deficits, instead implicating policy environments that reward volume-based sales over service-oriented care (23). Externally, this contrasts with successful enforcement models in higher-income settings, where digital systems reduced non-prescription sales by over 20% (19), but in Jordan, sporadic inspections sustain a “paper laws” culture, as described qualitatively. Acknowledging these challenges compels a shift from pharmacist-blame to system-reform, recognizing that without economic realignment (consultation fees), stewardship remains aspirational rather than actionable.

To bridge the knowledge-practice gap, multifaceted reforms are imperative. Digital prescription platforms could enhance traceability, addressing qualitative calls for accountability while mitigating “pharmacy shopping” (24). Mandatory AMS-integrated continuing professional development (CPD), linked to licensure, would build resilience against pressures, as supported by our training's protective role (25). Economic incentives must decouple income from sales, piloting reimbursement for counseling to align business viability with public health (23). Interprofessional collaboration, via shared guidelines and communication channels, could harmonize messaging, reducing patient-driven conflicts (26–30). Public campaigns to recalibrate antibiotic norms are equally vital, targeting cultural expectations that fuel demand.

While this study foregrounds the perspectives of community pharmacists, these perspectives reflect the operation of broader structural forces rather than isolated professional behavior. National health insurance coverage gaps, limited physician accessibility—particularly in rural and underserved areas—competitive retail pharmacy markets, and prevailing regulatory culture collectively shape patient demand and pharmacist decision-making. In contexts where physician consultations are costly or inaccessible, pharmacies increasingly function as de facto primary care entry points, intensifying pressure to dispense antibiotics without prescriptions. Similarly, fragmented insurance coverage and price-sensitive markets reinforce volume-based dispensing models, constraining pharmacists' ability to prioritize stewardship over economic survival. Although these macro-level determinants were not directly measured, they emerged consistently through qualitative narratives and are integral to interpreting pharmacy-level behavior as a manifestation of system-level constraints rather than individual practice failure.

Translating these policy priorities into practice in Jordan, however, requires context-sensitive and phased implementation strategies. Importantly, many proposed interventions can be integrated within existing institutional and regulatory frameworks rather than relying on new funding streams. Digital prescription verification could be introduced incrementally through national e-health infrastructures overseen by the Ministry of Health, including systems already used for insurance coverage and prescription tracking. Similarly, AMS-focused CPD training could be embedded within existing pharmacist licensing and renewal requirements through collaboration between the Ministry of Health, the Jordan Pharmacists Association, and academic institutions. At the same time, the economic implications for community pharmacies must be explicitly acknowledged. Abrupt enforcement of prescription-only regulations may disproportionately affect independent pharmacies operating in highly competitive markets. A phased enforcement strategy, combined with consistent nationwide inspections, could reduce competitive disadvantage by ensuring uniform compliance across pharmacies and, over time, limit customer loss by reducing pharmacy shopping and reshaping patient expectations. From a regulatory standpoint, incremental implementation aligned with Jordan's National Action Plan on Antimicrobial Resistance offers a realistic and policy-consistent pathway, with pilot digital systems, targeted inspections in high-risk areas, and gradual integration of stewardship indicators into pharmacy audits representing feasible starting points.

Future research should evaluate these interventions longitudinally, incorporating objective metrics like dispensing audits to link behaviors to resistance trends. By challenging education-centric assumptions and emphasizing systemic alignment, our model offers a roadmap for LMICs, transforming pharmacists from AMR contributors to stewards in mitigating this escalating threat.

## 5 Conclusion

This mixed-methods study reveals that while community pharmacists in Jordan possess strong knowledge and favorable attitudes toward antimicrobial stewardship, they remain constrained by structural, economic, and sociocultural pressures that perpetuate non-prescription antibiotic dispensing. The findings underscore that stewardship success depends less on awareness than on the systems that enable or inhibit its practice. Weak enforcement, profit-driven business models, and patient expectations collectively sustain behaviors that undermine national and global efforts to contain antimicrobial resistance.

Addressing these entrenched barriers requires coordinated reform that aligns incentives, accountability, and public education. Implementing digital prescription verification, strengthening Ministry of Health inspection capacity, and embedding antimicrobial stewardship into continuing professional development would provide pharmacists with both the authority and infrastructure to act as effective stewards. At the same time, sustained community engagement and collaboration among pharmacists, physicians, and other healthcare providers are essential to recalibrate public expectations around antibiotic use.

By integrating behavioral, regulatory, and economic perspectives, this study provides a framework for designing context-appropriate antimicrobial stewardship policies in low- and middle-income countries. Translating these insights into action will be critical for reducing inappropriate antibiotic use and safeguarding the effectiveness of antimicrobials for future generations.

## 6 Limitations

This study has several limitations that should be acknowledged when interpreting its findings. First, the cross-sectional design captures perceptions and practices at a single point in time, which restricts causal inference and limits the ability to assess changes in dispensing behavior or policy impact over time. Future longitudinal or quasi-experimental designs would be valuable to determine whether the identified predictors of inappropriate antibiotic dispensing remain stable following regulatory or educational interventions.

Second, both the survey and interview components relied on self-reported data, which are inherently susceptible to recall and social desirability biases. Pharmacists may have overstated their adherence to antimicrobial stewardship (AMS) principles or underreported non-prescription dispensing to align with perceived professional norms. Nevertheless, the mixed-methods design and anonymity of responses likely mitigated some of these effects by encouraging candid participation and allowing cross-validation between quantitative and qualitative findings.

Third, although the study included participants from five major Jordanian governorates, the sample may not fully represent all community pharmacists across the country, particularly those in remote or underserved regions where regulatory oversight and patient behavior could differ. Similarly, the qualitative sample, while diverse and saturated, may not have captured every contextual nuance influencing antibiotic dispensing. Broader national sampling and inclusion of rural pharmacies could provide a more comprehensive perspective in future research.

Fourth, while the study examined perceived barriers and determinants, it did not directly measure antibiotic sales volume or link dispensing behavior to clinical outcomes or resistance patterns. Integrating such objective indicators—through pharmacy audit data or antimicrobial consumption databases—would enrich future analyses and provide stronger evidence of stewardship impact. Although economic pressure emerged as a prominent perceived influence, the study did not quantify pharmacy income dependency, antibiotic profit margins, or financial vulnerability, and economic findings should therefore be interpreted as contextual rather than causal. These factors were explored qualitatively to capture contextual decision-making processes. Future research should incorporate objective financial indicators or economic modeling to better quantify the relationship between pharmacy economics and antimicrobial stewardship behavior.

Finally, as with all self-administered online surveys, there is a potential for non-response bias, as pharmacists more interested in AMS issues may have been disproportionately likely to participate. However, the achieved sample size exceeded the calculated minimum and demonstrated wide demographic and practice variation, supporting the credibility and generalizability of the findings within the study context.

Despite these limitations, this investigation provides a detailed mixed-methods assessments of AMS barriers among community pharmacists in Jordan to date. Its integration of quantitative predictors with qualitative insights provides a robust foundation for policy design, targeted interventions, and future research aimed at strengthening antimicrobial stewardship in similar low- and middle-income settings.

## Data Availability

The original contributions presented in the study are included in the article/supplementary material, further inquiries can be directed to the corresponding author.
